# Peptides derived from the integrin β cytoplasmic tails inhibit angiogenesis

**DOI:** 10.1186/s12964-018-0248-8

**Published:** 2018-07-03

**Authors:** Zhongyuan Cao, Xinfeng Suo, Yudan Chu, Zhou Xu, Yun Bao, Chunxiao Miao, Wenfeng Deng, Kaijun Mao, Juan Gao, Zhen Xu, Yan-Qing Ma

**Affiliations:** 1 School of Environmental and Chemical Engineering, Shanghai University, Shanghai, China; 2 School of Life Sciences, Shanghai University, Shanghai, China; 3 Blood Research Institute, Blood Center of Wisconsin, part of Versiti, 8727 Watertown Plank Rd, Milwaukee, WI 53226 USA

**Keywords:** Angiogenesis, Endothelial cells, Integrins, Cytoplasmic tails, β3-endonexin

## Abstract

**Background:**

Integrins are essential regulators of angiogenesis. However, the antiangiogenic potential of peptides derived from the integrin cytoplasmic tails (CT) remains mostly undetermined.

**Methods:**

Here we designed a panel of membrane-penetrating peptides (termed as mβCTPs), each comprising a C-terminal NxxY motif from one of the conserved integrin β CTs, and evaluated their antiangiogenic ability using both in vitro and in vivo approaches.

**Results:**

We found that mβ3CTP, mβ5CTP and mβ6CTP, derived respectively from the integrin β3, β5 and β6 CTs, but not others, exhibit antiangiogenic ability. Interestingly, we observed that the integrin β3, β5 and β6 CTs but not others are able to interact with β_3_-endonexin. In addition, the antiangiogenic core in mβ3CTP is identical to a previously identified β_3_-endonexin binding region in the integrin β3 CT, indicating that the antiangiogenic mβCTPs may function via their binding to β_3_-endonexin. Consistently, knockdown of endogenous β_3_-endonexin in HUVECs significantly suppresses tube formation, suggesting that β_3_-endonexin is proangiogenic. However, neither treatment with the antiangiogenic mβCTPs nor knockdown of endogenous β_3_-endonexin affects integrin-mediated HUVEC adhesion and migration, indicating that their antiangiogenic effect may not rely on directly regulating integrin activity. Importantly, both treatment with the antiangiogenic mβCTPs and knockdown of endogenous β_3_-endonexin in HUVECs inhibit VEGF expression and cell proliferation, thereby providing mechanistic explanations for the functional consequences.

**Conclusion:**

Our results suggest that the antiangiogenic mβCTPs can interact with β_3_-endonexin in vascular endothelial cells and suppress its function in regulating VEGF expression and cell proliferation, thus disclosing a unique pathway that may be useful for developing novel antiangiogenic strategies.

**Electronic supplementary material:**

The online version of this article (10.1186/s12964-018-0248-8) contains supplementary material, which is available to authorized users.

## Background

Integrins are a family of cell adhesion receptors that primarily support cell interaction with extracellular matrix, thus being able to mediate cell adhesion and migration [1, 2]. The integrins consist of non-covalently associated α/β subunits and are widely expressed. Among the integrin members expressed in vascular endothelial cells, αvβ3 has been extensively investigated and well recognized as an antiangiogenic target [3–5]. It has been found that integrin αvβ3 is prominently expressed in angiogenic vasculature and targetable to induce apoptosis of proliferative tumor endothelial cells [6–8], and these findings have sparked the antitumor stratagems by developing specific inhibitors that block αvβ3-ligand interactions. Practically, the integrin αvβ3 antagonisms with either functional blocking antibodies or ligand-mimetic peptides have shown promising antiangiogenic and antitumor effects [9–11]. However, substantial controversies have arisen due to some inconsistent observations. First, enhanced tumor growth can be observed in integrin β3-deficient mice [12]. Second, low concentrations of RGD-mimetic integrin αvβ3 inhibitors unexpectedly promote tumor growth and angiogenesis in mice [13, 14]. Adding further to these uncertainties is a study from Merck that Cilengitide, a highly anticipated integrin αvβ3 antagonist, has failed its first Phase III clinical trial on treating glioblastoma [15]. Hence, these inconsistencies suggest that more studies are required in order to further optimize the therapeutic outcomes.

The integrin α/β cytoplasmic tails (CTs) are typically short (except for β4) and lack intrinsic enzymatic activity, but they, especially the integrin β CTs, play indispensable roles in the regulation of integrin bidirectional signaling via interacting with their cytoplasmic binding partners [16, 17]. Each of the integrin β CTs (except for β4 and β8) encompasses two conserved NxxY motifs, which constitute essential hubs for recruiting the β CT binding proteins. For example, the membrane-proximal NxxY motif interacts with talin, a key integrin activator; and the C-terminal NxxY motif specifically binds kindlins, a family of essential integrin co-activators [18–20]. In addition, the C-terminal NxxY motif NITY in the β3 CT is also involved in interacting with β3-endonexin [21, 22]. The kindlin family members (kindlin-1, − 2 and − 3) essentially support integrin-mediated cell adhesion and migration, and deficiency of any one of them leads to tissue-specific integrin dysfunctions [23]. It has been shown that the polypeptide β3-endonexin can selectively interact with the integrin β3 CT but not the β1 and β2 CTs [21, 22], but it still inconclusive if β3-endonexin plays a role in regulating β3-integrin activation [20, 22].

Kindlins and β3-endonexin are both expressed in vascular endothelial cells [20, 24–27]. Kindlin-2 has been shown to support integrin αvβ3-mediated endothelial cell adhesion and migration and promote angiogenesis in zebrafish and mice [20, 26]. Interestingly, β3-endonexin was ever reported to be able to negatively regulate the angiogenic response under hypoxic conditions [24]. Since both kindlins and β3-endonexin recognize the C-terminal NITY motif of the integrin β3 CT, we hypothesize that the peptides comprising the NITY motif or its conserved counterparts in the other integrin β CTs may have the ability to regulate angiogenesis. To test this posibility, we employ six membrane-penetrating peptides derived from the conserved integrin β CTs, each embodying a C-terminal NxxY motif from one of the β CTs, and evaluate their angiogenic capacity using in vitro and in vivo approaches, and finally disclose an important functional relationship between the integrin β CT peptides and β3-endonexin in regulating angiogenesis.

## Methods

### Proteins and peptides

The GST-fused integrin β CT proteins were expressed in *E. coli* BL21 (DE3) and purified by affinity chromatography of glutathione. All the integrin derived peptides used in this study were synthesized and purified to more than 98%. The peptides were dissolved in DMSO at 100 μM concentration and further diluted into culture medium to final concentrations when treating cells.

### Cell culture and transfection

Primary human umbilical vein endothelial cells (HUVECs) were seeded in fibronectin coated tissue culture dishes and cultured in MCDB medium (Sigma) supplemented with 15% FBS, endothelial cell growth supplements (Sigma) and heparin. Murine RM1 prostate cancer cells were cultured in DMEM/F12 medium (Hyclone) supplemented with 10% FBS. All these cultures were maintained at 37 °C in a humidified tissue culture incubator with an atmosphere of 5% CO_2_. Targefect reagents (Targeting Systems) were used to transfect HUVECs with siRNA duplex or β3-endonexin plasmid for knocking down endogenous β3-endonexin or overexpressing exogenous β3-endonexin in HUVECs.

### In vitro HUVEC tube formation

24-well tissue culture plates were pre-coated with 300 μl per well of growth factor-reduced Matrigel (BD Biosciences). HUVECs (0.25 × 10^5^/well) were seeded on the polymerized Matrigel in 3× diluted culture medium with or without the indicated mβCTPs (20 μM at a final concentration). After 8–12 h of incubation, capillary-like tube structures formed by HUVECs were examined under an inverted microscope.

### In vivo Matrigel plug assay

Aliquots of liquid Matrigel (BD Biosciences) supplemented with 600 ng/ml of bFGF and 100 U/ml of heparin were mixed with the indicated mβCTPs (50 μM at final concentration) and injected into BALB/c nude mice subcutaneously. Matrigel mixture without the peptides was used as a control. Matrigel solution can quickly polymerize to form a solid plug after subcutaneous injection. 7 days after the injection, the Matrigel plugs were carefully harvested from mice for quantification of hemoglobin and histological staining.

### Solid tumor growth model

In vivo tumor growth was achieved by subcutaneously implanting murine RM1 prostate cancer cells into BALB/c nude male mice (1.2 × 10^6^ cells per mouse). Starting from day 5 after cancer cell inoculation, mβCTPs or saline alone was intratumorally administrated every other day (50 μM at a final concentration, 100 μl per mouse). Meanwhile, tumor volumes (v) were determined by measuring the length (l) and the width (w) and calculated using the following formula: v = 0.52(l × w^2^). Eventually, the tumor tissues were carefully isolated and processed for histological studies.

### HUVEC proliferation analysis

Cell proliferation was measured by the CCK-8 Cell Counting Kit (Dojindo) according to the manufacturer’s instruction. In brief, HUVECs were seeded in 96-well plates at a density of 8 × 10^3^/well and treated with the indicated peptides for 48 h under culture conditions. After that, cells were incubated with the CCK-8 solution for 3 h and the absorbance at 450 nm was measured using a microplate reader. For some experiments, the MTT assay (Sigma) was also performed to confirm the results.

### HUVEC adhesion and migration

HUVECs were pretreated with the tested peptides for 2 h and their effects on cell adhesion and migration were evaluated as we previously described [20]. For measuring HUVEC adhesion, HUVECs were allowed to incubate with immobilized fibrinogen or vitronectin in slide chambers in the presence or absence of VEGF (20 ng/ml) for 30 min at 37 °C. The suspended cells were washed away and the adherent cells were fixed by 70% methanol and stained with 1% toluidine blue for visualization. HUVEC migration was evaluated using Transwell plates with 8 μm pore size. HUVECs were added to the upper chamber that was precoated with either fibrinogen or vitronectin and allowed to migrate for 8 h in the presence or absence of VEGF (20 ng/ml) in the lower chamber. After migration, the cells on the upper surface of the chamber were removed and the migrated cells on the bottom surface were fixed with methanol and stained with 1% toluidine blue for performing microscopic cell counts.

### Yeast two-hybrid

To test the interaction of β3-endonexin or kindlin-2 with the integrin β CTs, the Matchmaker™ Gold Yeast Two-hybrid system (Clontech) was employed as we previously described [28]. In brief, β3-endonexin or kindlin-2 was cloned into vector pGBKT7, and the integrin β CTs were cloned into vector pGADT7. Self-activation of these constructs was evaluated and excluded. Protein-protein interaction was examined according to the manufacturer’s protocol.

### Pull-down assays

HUVECs transiently expressing EGFP-β3-endonexin were lysed using CelLytic Cell Lysis Reagent (Sigma) in the presence of protease inhibitors (Roche). Purified GST and GST-fused integrin β CT proteins were loaded on Glutathione Sepharose 4B beads (GE Healthcare) and incubated with the lysates of HUVECs for overnight in a 4 °C cold room in the presence or absence of the indicated mβCTPs. After washing, EGFP-β3-endonexin and endogenous kindlin-2 in the precipitates were analyzed by Western blotting. GST proteins loaded on the beads were evaluated by Coomassie blue staining.

### Knockdown of endogenous β3-endonexin in HUVECs

An siRNA duplex specifically targeting β3-endonexin was used to knocking down endogenous β3-endonexin (RiboBio). The sequence targeting β3-endonexin was GAACTTGTCAAATGAGTCT. A non-targeting control siRNA duplex was used as a control. After transfecting HUVECs with siRNA duplexes for 48 h, cells were harvested for functional analysis. Meanwhile, the expressing levels of endogenous β3-endonexin were evaluated by quantitative RT-PCR.

### Quantitative RT-PCR (qRT-PCR)

Total RNA was extracted from HUVECs using a TRIzol kit (Invitrogen). A PrimeScript® RT reagent Kit (Takara) was used for cDNA synthesis. For quantitative analysis of the mRNA levels of the tested genes, the synthesized cDNA was used as a template and subjected to 40 cycles of quantitative PCR using Takara SYBR Premix Ex Taq™ in the CFX96™ Real-Time PCR Detection System (Bio-Rad). Primer sequences (forward and reverse) used in this study were as follows: β3-endonexin forward, 5’-TCACTGAAGTTGGATGGTCTGT-3′, and reverse, 5’-AGTCCATTTCTGTGCTTTTGCT-3′; VEGFA forward, 5’-AGGGCAGAATCATCACGAAGT-3′, and reverse, 5’-AGGGTCTCGATTGGATGGCA-3′; β-actin forward, 5’-TCCATCATGAAGTGTGACGT-3′, and reverse, 5′- AGGAGGAGCAATGATCTTGA-3′. For each independent experiment, samples were loaded in triplicate, and the value of each sample was normalized by β-actin.

## Results

### The mβCTPs selectively inhibit HUVEC tube formation

**Fig. 1:**
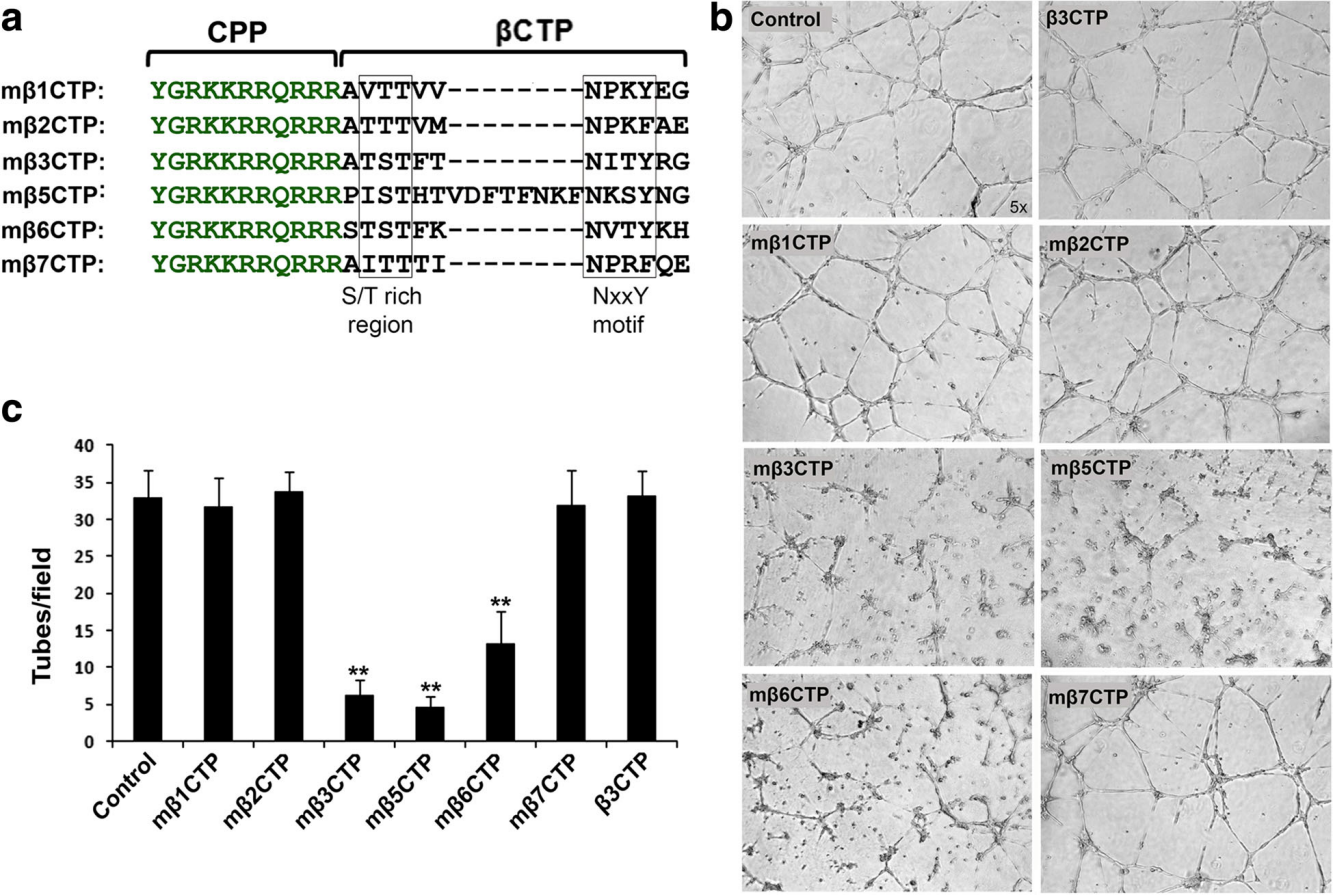
The mβCTPs selectively inhibit HUVEC tube formation. **a** Amino-acid sequences of mβCTPs. In each of mβCTPs, a cell-penetrating peptide (CPP) was fused with a short amino-acid sequence from one of the C-termini of integrin β CTs (βCTP), comprising an NxxY/F motif and a proximal S/T cluster. **b** HUVECs were seeded on polymerized Matrigel with or without the indicated mβCTPs (20 μM at a final concentration) for 8 h. The formed capillary-like tube structures were recorded under an inverted microscope (5× objective). **c** The capillary-like polygon tubes were quantified. Results were expressed as means ±SD of three or more experiments, and statistical significance was analyzed using Student’s *t* test (**, *p* < 0.01)

The C-termini of the integrin β CTs (except for β4 and β8) contain a conserved NxxY/F motif and an S/T cluster region (Fig. 1a), which are important docking regions for cytoplasmic binding partners, such as kindlins and β3-endonexin. Since it has been reported that kindlins and β3-endonexin are both involved in regulating angiogenesis, we sought to evaluate the antiangiogenic capacity of the C-terminal region of the β CTs. As shown in Fig. 1a, the C-terminal regions of the β CTs comprising the NxxY/F motif and the S/T cluster (βCTP) were selected and fused with a cell penetrating TAT amino-acid sequence [29, 30], thus to constitute membrane-permeable peptides (termed as mβCTP) (also see Additional file 1: Figure S1). HUVEC tube formation assays were performed to functionally analyze angiogenesis in vitro. HUVECs were seeded on polymerized Matrigel to induce the formation of capillary-like tube structures. As shown in Fig. 1b, c, treating HUVECs with mβ3CTP, mβ5CTP and mβ6CTP substantially impaired HUVEC tube formation. However, the other mβCTPs, including mβ1CTP, mβ2CTP and mβ7CTP, had no effect on inhibiting HUVEC tube formation. As a control, β3CTP lacking the cell penetrating ability failed to inhibit HUVEC tube formation (Fig. 1b and Additional file 1: Figure S1b). Together, these results suggest that different mβCTPs have distinct antiangiogenic ability.

### The mβCTPs selectively inhibit angiogenesis in subcutaneous Matrigel plugs

**Fig. 2:**
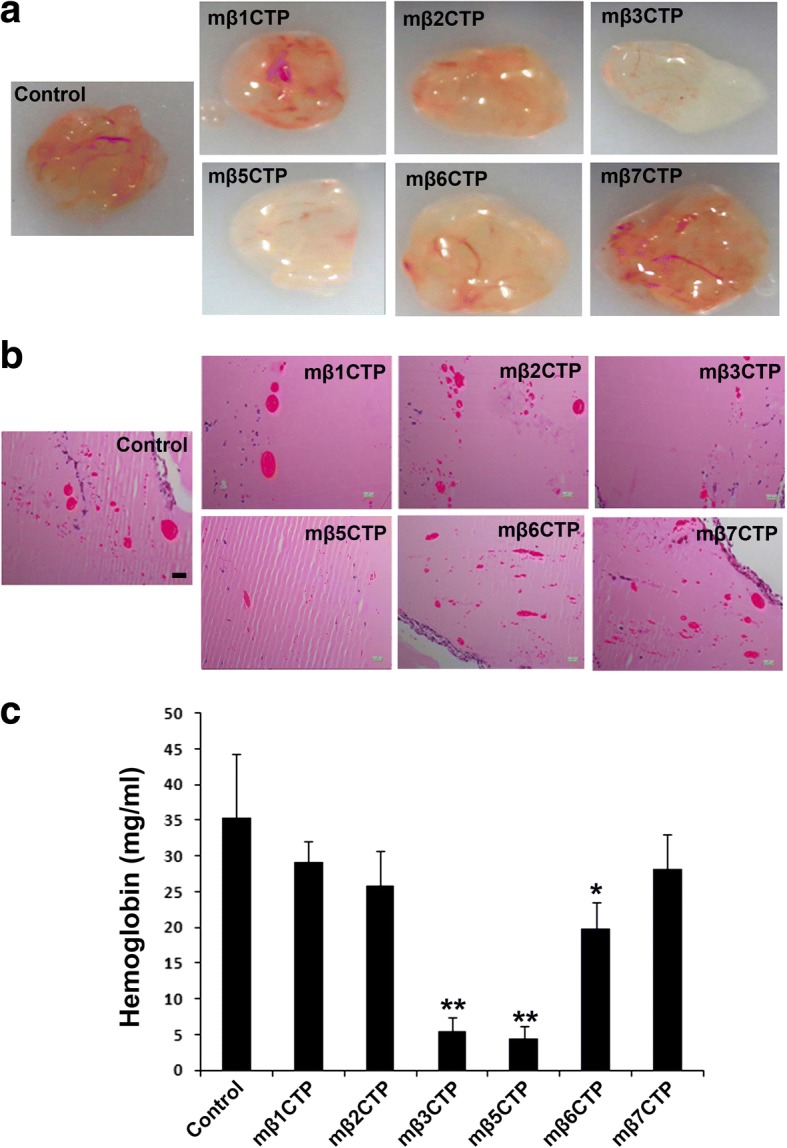
The mβCTPs selectively suppress blood vessel formation in implanted Matrigel plugs. **a** Aliquots of Matrigel liquid supplemented with the indicated mβCTPs (50 μM at a final concentration) were subcutaneously injected into BALB/c nude mice (*n* = 5) to form solid Matrigel plugs. Matrigel that did not contain peptides was used as a control. After 7 days, Matrigel plugs were carefully isolated and photographed. **b** Matrigel plugs were fixed and paraffin-embedded for histological analysis. Representative H&E staining images of the Matrigel plug sections were shown. Scale bar, 200 μm. **c** The amount of hemoglobin in Matrigel plugs was quantified by Drakin’s reagent. Results were expressed as means ± SD of five samples; statistical significance was analyzed using Student’s *t* test (*, *p* < 0.05; **, *p* < 0.01)

We next employed the in vivo Matrigel plug assay to evaluate the antiangiogenic ability of the mβCTPs. Liquid Matrigel can be quickly solidified to form a plug after subcutaneous injection in mice. As shown in Fig. 2a, the implanted Matrigel plug could effectively trigger angiogenesis to form visible blood vessels (see control). Histological analysis by H&E staining revealed that the newly formed vessels were randomly distributed in implanted Matrigel plugs (Fig. 2b, control). Due to the significant variation of the sizes of formed vessels, we decided to evaluate the vascularization by quantifying hemoglobin contained in the Matrigel plugs (Fig. 2c). We found that three mβCTPs (mβ3CTP, mβ5CTP and mβ6CTP) that could suppress HUVEC tube formation in vitro could also inhibit blood vessel formation in Matrigel plugs in vivo, although mβ6CTP was less effective. However, the formation of blood vessels and the amount of hemoglobin in Matrigel plugs after treatment with mβ1CTP, mβ2CTP and mβ7CTP were similar with the non-treated control. These results further verify the distinct roles of different mβCTPs in regulating angiogenesis.

### The antiangiogenic mβCTPs suppress solid tumor growth

**Fig. 3:**
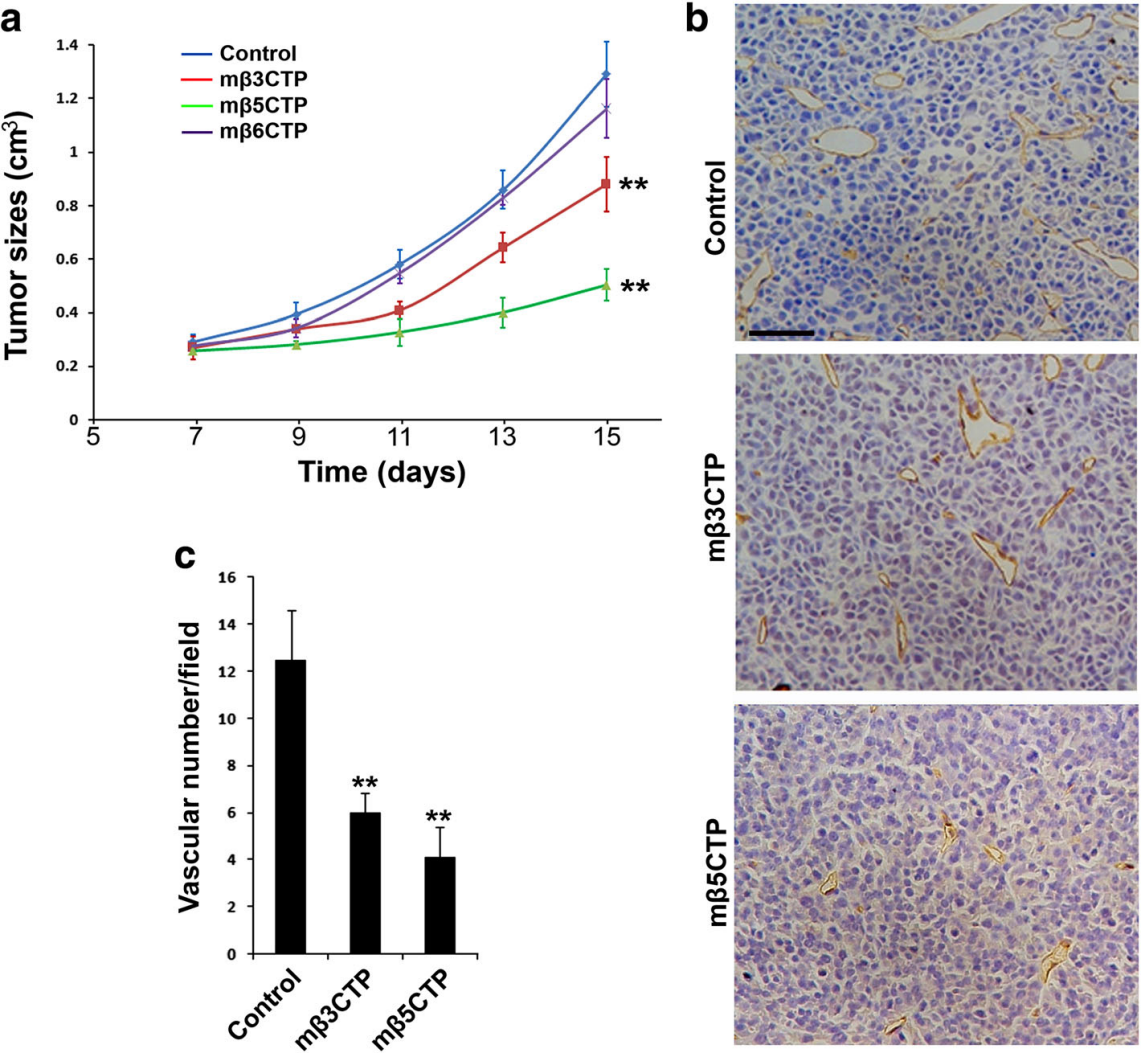
The antiangiogenic mβCTPs show antitumor activity. **a** 1.2 × 10^6^ of RM1 cancer cells were subcutaneously injected into BALB/c nude mice (*n* = 6). Starting on day 5, the formed tumor areas were subjected to treatment by local injection of 100 μl of the indicated mβCTP solution (50 μM at a final concentration) every other day. PBS alone was used as a control. Length (l) and width (w) of the formed tumors were measured, and tumor volumes (v) were calculated by using the following formula: v = 0.52(l × w^2^). **b, c** The tumor tissues were harvested on day 15 and subjected to IHC staining for CD31, a marker of vascular endothelial cells. Blood vessels in solid tumor tissues were quantified. Scale bar, 50 μm. The results were expressed as means ±SD; statistical significance was analyzed using Student’s *t* test (**, *p* < 0.01)

Angiogenesis plays an essential role for supporting in vivo solid tumor growth. Therefore, we tested if the antiangiogenic mβCTPs identified above have antitumor capacity. RM1 prostate tumor cells were subcutaneously injected into BALB/c nude mice and allowed to grow to solid tumors. When the size of tumors reached to ~ 200 mm^3^ (around day 5 after inoculation), tumor-bearing mice were locally treated with the antiangiogenic mβCTPs. Compared to a sham treatment in control mice, tumor growth was significantly reduced in mice treated with either mβ3CTP or mβ5CTP (Fig. 3a and Additional file 1: Figure S3). However, under the same experimental conditions, treatment with mβ6CTP failed to suppress tumor growth, which may be due to its weaker antiangiogenic ability (Fig. 2). These results demonstrate that these three antiangiogenic mβCTPs (mβ3CTP, mβ5CTP and mβ6CTP) have different antitumor capacity.

To explore if the antitumor function of mβ3CTP and mβ5CTP correlates with their antiangiogenic property, the formed blood vessels in tumor tissues were analyzed by histological staining of CD31, a marker of vascular endothelial cells. As shown in Fig. 3b, c, after treatment with mβ3CTP or mβ5CTP, the blood vessel numbers in tumors were significantly less than those in control tumors with sham treatment, demonstrating that the antitumor property of mβ3CTP and mβ5CTP can be ascribed to their antiangiogenic ability. To test if the antiangiogenic peptides also inhibit tumor cell proliferation, we treated the RM1 prostate tumor cells with either mβ3CTP or mβ5CTP under culture conditions and found that neither had significant effect at the experimental conditions (Additional file 1: Figure S2). Collectively, these results suggest that mβ3CTP and mβ5CTP may suppress in vivo solid tumor growth via their antiangiogenic function.

### The antiangiogenic mβCTPs do not affect HUVEC adhesion and migration under stimulations

**Fig. 4:**
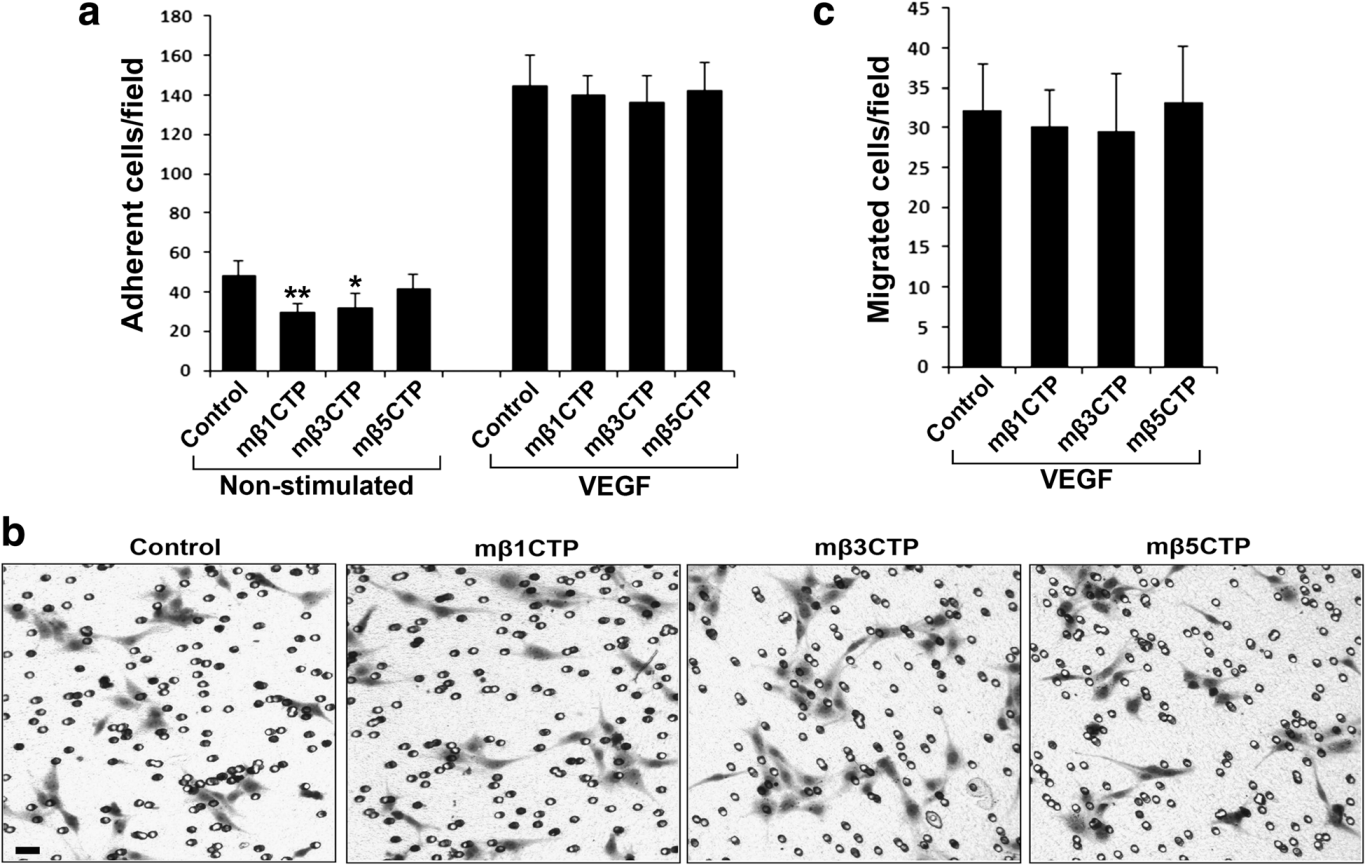
The antiangiogenic mβCTPs fail to affect VEGF-induced HUVEC adhesion and migration. **a** HUVECs were treated with the indicated mβCTPs (20 μM) and allowed to adhere to immobilized fibrinogen for 30 min in the absence or presence of 20 ng/ml of VEGF. The adherent cells were fixed, stained and counted as described in methods. **b, c** HUVECs were treated with the indicated mβCTPs (20 μM) and allowed to migrate to Transwell membrane coated with fibrinogen for 8 h in the presence of VEGF (20 ng/ml). The migrated cells were fixed, stained, photographed and counted. Scale bar, 20 μm. Results were expressed as means ± SD of five samples; statistical significance was analyzed using Student’s *t* test (*, *p* < 0.05; **, *p* < 0.01)

Since these antiangiogenic mβCTPs are integrin β CT based peptides, we next sought to evaluate if they affect integrin (especially αvβ3) mediated HUVEC adhesion and migration on fibrinogen. Under the same dose as used in the HUVEC tube formation assay, we detected that mβ3CTP could moderately suppress HUVEC adhesion to immobilized fibrinogen in the absence stimulation while mβ5CTP had no effect (Fig. 4a). However, mβ1CTP, one of the non-antiangiogenic mβCTPs, also inhibited HUVEC adhesion under the same condition. Interestingly, none of the mβCTPs affects VEGF-stimulated HUVEC adhesion (Fig. 4a). In addition, we found that all the mβCTPs failed to affect VEGF-induced HUVEC migration on fibrinogen (Fig. 4b, c). We also performed these assays on vitronectin and obtained the similar results (Additional file 1: Figure S4). Since HUVECs poorly migrated in the absence of stimulation (results not shown), we did not evaluate the effect of mβCTPs on HUVEC migration under non-stimulated conditions. Together, these results show that the antiangiogenic ability of the mβCTPs is not consistent with their capacity to affect integrin activity, indicating that the antiangiogenic mβCTPs may employ integrin-independent mechanisms to modulate angiogenesis.

### Identification of the antiangiogenic core in mβ3CTP

**Fig. 5:**
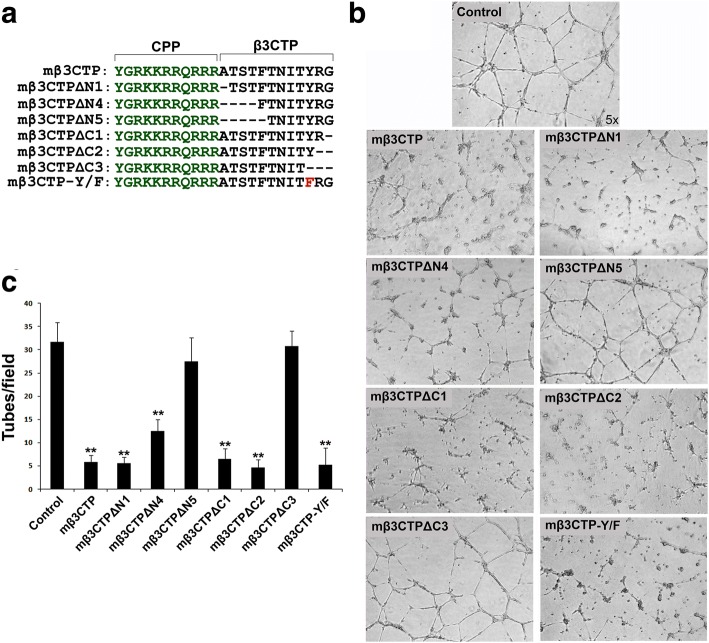
Identify the antiangiogenic core region in mβ3CTP. **a** The amino-acid sequences of different mβ3CTP variants with sequential residue deletions from either side of β3CTP were shown. **b, c** HUVECs were seeded on polymerized Matrigel in 24-well plates and treated with the indicated mβ3CTP variants (20 μM at a final concentration) or PBS alone as a control. The formed capillary-like structures were counted under an inverted microscope (5× objective). Results were expressed as means ±SD from three experiments; statistical significance was analyzed using Student’s *t* test (**, *p* < 0.01)

Next, we attempted to map the antiangiogenic core region in mβ3CTP. To do so, a series of truncated mβ3CTP variants with sequential deletions from either side of the β3CTP were prepared (Fig. 5a). Then the antiangiogenic ability of each of them was tested using the HUVEC tube formation assay. As shown in Fig. 5b, c, deletion of the C-terminal RG residues in mβ3CTP did not affect the antiangiogenic efficacy; however, deletion of the C-terminal YRG residues that includes tyrosine in the NITY motif abrogated the antiangiogenic ability, indicating that the NITY motif is required for the antiangiogenic function. In addition, mβ3CTP missing the N-terminal TST region showed a significantly compromised antiangiogenic ability, suggesting that the conserved S/T cluster in mβ3CTP is also involved in supporting antiangiogenesis. Particularly, mβ3CTP with deletion of one more residue after the TST cluster totally lost the antiangiogenic ability, implying that the N-terminally flanking residues of the NITY motif also participate in antiangiogenesis. Together, these results suggest that the xNITY motif (here x represents two or more residues) in mβ3CTP is primarily responsible for the antiangiogenic function.

Since the residue tyrosine in the NITY motif of the β3 CT is a phosphorylation site [31, 32], we next evaluated the potential effect of tyrosine phosphorylation on the antiangiogenic ability of mβ3CTP. As shown in Fig. 5a, a structurally conserved Y/F mutation was introduced into mβ3CTP to prevent tyrosine phosphorylation. Notably, the mβ3CTP variant carrying the Y/F mutation still sufficiently inhibited HUVEC tube formation as shown in Fig. 5b, c, demonstrating that tyrosine phosphorylation in the NITY motif is not required for mβ3CTP to suppress angiogenesis. In addition, we employed a modified mβ3CTP with a phosphorylated tyrosine and found that such a tyrosine phosphorylated peptide showed a significantly reduced ability in inhibiting HUVEC tube formation (data not shown). Together, these results indicate that tyrosine phosphorylation in mβ3CTP is not required for antiangiogenesis.

### The integrin β CTs and the derived mβCTPs selectively interact with β3-endonexin

**Fig. 6:**
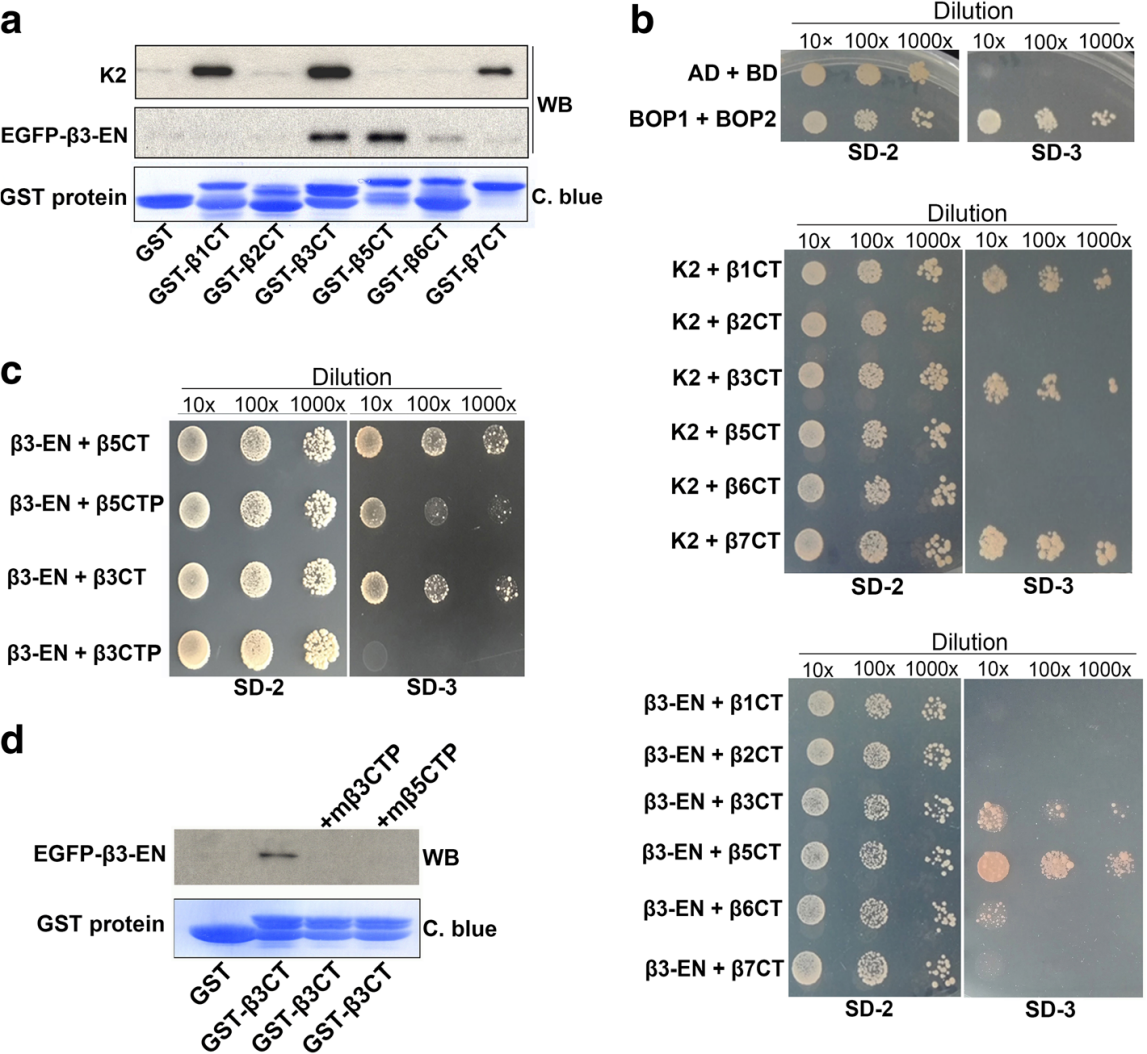
The antiangiogenic mβCTPs specifically interact with β3-endonexin. **a** Lysate aliquots of HUVECs exogenously expressing EGFP-β3-endonexin (EGFP-β3-EN) were incubated with purified GST or GST-fused β CT proteins coupled on Glutathione Sepharose beads. After incubation, the beads were washed and the precipitated proteins were separated by SDS-PAGE. The loaded GST proteins on the beads were assessed by Coomassie blue (C. blue) staining. The co-precipitated EGFP-β3-endonexin and endogenous kindlin-2 (K2) were detected by Western blotting. **b** Interaction of the β CTs with β3-EN or K2 was evaluated using the yeast 2-hybrid system by a serial dilution method on selection media. Two known interacting molecules (Bop1 and Bop2) were employed here as a positive control, and empty vectors were transformed to serve as a negative control. The growth of yeast cells on SD-2 media (−Leu/−Trp) indicates a successful transformation; the growth on SD-3 selection media (−Leu/−Trp/-His) indicates a positive protein-protein interaction. **c** The interaction of β3-EN with β3CTP and β5CTP was evaluated using the yeast 2-hybrid system. **d** Interaction between EGFP-β3-endonexin and GST-β3 CT was evaluated by a pull-down assay in the presence of mβ3CTP or mβ5CTP (20 μM at a final concentration) as described in **a**

Multiple binding partners have been identified for the C-terminal NITY motif in the integrin β3 CT (details in the section of Discussion). Among them, β3-endonexin is specific for the β3 CT because it does not bind the β1 and β2 CTs [21, 22]. As summarized in Table 1, the NITY motif in the β3 CT is essentially required for interacting with β3-endonexin. Interestingly, the N-terminal residues beyond the NITY motif also participate in the interaction, which is compatible with the antiangiogenic core (xNITY) identified in mβ3CTP. Therefore, we hypothesize that β3-endonexin may serve as a functional effector for the antiangiogenic mβCTPs. To demonstrate this hypothesis, we first performed a pull-down experiment, in which the lysates of HUVECs expressing of EGFP-β3-endonexin were used to incubate with GST-fused integrin β CTs in the presence of glutathione-Sepharose beads. As shown in Fig. 6a, we found that EGFP-β3-endonexin was able to interact with both the β3 and β5 CTs. A relatively weak interaction between EGFP-β3-endonexin and the β6 CT was also detected. However, the signal of interaction between EGFP-β3-endonexin and the β1, β2 or β7 CT was close to the background. Interestingly, endogenous kindlin-2 was found to be able to bind the β1, β3 and β7 CTs but not others, showing that kindlin-2 and β3-endonexin have distinct binding patterns to the integrin β CTs.

**Table 1:** Summary of the yeast two-hybrid results from previous publications [21, 22]

Name	Amino-acid sequence	β3-EN binding
β1CT:	-KLLMIIHDRREFAKFEKEKMNAKWDTGENPIYKSAVTTVVNPKYEGK	-^*(a)*^
β2CT:	-KALIHLSDLREYRRFEKEKLKSQWN-NDNPLFKSATTTVMNPKFAES	-
β3CT:	-**KLLITIHDRKEFAKFEEERARAKWDTANNPLYKEATSTFTNITYRGT**^*(c)*^	+
β3CT-Δ762:	-**KLLITIHDRKEFAKFEEERARAKWDTANNPLYKEATSTFTNITYRG**	+
β3CT-Δ761:	-**KLLITIHDRKEFAKFEEERARAKWDTANNPLYKEATSTFTNITYR**	+
β3CT-Δ760:	-**KLLITIHDRKEFAKFEEERARAKWDTANNPLYKEATSTFTNITY**	+
β3CT-Δ759:	-**KLLITIHDRKEFAKFEEERARAKWDTANNPLYKEATSTFTNIT**	-
β3CT-Δ755:	-**KLLITIHDRKEFAKFEEERARAKWDTANNPLYKEATSTF**	-
αIIb/β3CT:	-KVGFFKRNRPPLEEDDEEGQ**NITYRGT**	-
β1/β3CT1:	-KLLMIIHDRREFAKFEKEKMNAKWDTGENPIYKSAVTTVV**NITYRGT**	+
β1/β3CT2:	-KLLMIIHDRREFAKFEKEKMNAKWDTGENPIYKSAVTTVV**NITY**EGK	+
β3/β1CT1:	-**KLLITIHDRKEFAKFEEERARAKWDTANNPLYKEATSTFT**NPKYEGK	-
β3/β1CT2:	-**KLLITIHDRKEFAKFEEERARAKWDTANNPLYKEATSTFT**NPKY**RGT**	-
β3CT-I/P:	-**KLLITIHDRKEFAKFEEERARAKWDTANNPLYKEATSTFTN**P**TYRGT**	-
β3CT-Y/F:	-**KLLITIHDRKEFAKFEEERARAKWDTANNPLYKEATSTFTNIT**F**RGT**	+
Binding core:		x**NITY**^*(b)*^

^(a)^The interactions between β3-endonexin (β3-EN) and the original and chimeric integrin β CTs were defined by arbitrary units (AU) of β-Galactosidase as described in the publications. (Positive interactions (+): AU > 5; negative interactions (−): AU < 5)

^(b)^The “x” represented undetermined amino acids (number and residues)

^(c)^Amino acids from the integrin β3 CT were shown in boldface

Further, we employed the yeast 2-hybrid system to verify the interaction. As shown in Fig. 6b, both the integrin β3 and β5 CTs showed a significant binding signal to β3-endonexin; the integrin β6 CT exhibited a weak but detectable interaction with β3-endonexin; however, the interaction of β3-endonexin with the β1, β2 and β7 CTs was undetectable. Meanwhile, kindlin-2 was found to interact with the β1, β3 and β7 CTs but not with the other β CTs under the experiment conditions. These results are consistent with the observation from the pull down assays (Fig. 6a). In the yeast two-hybrid assay, empty vectors and a pair of known binding molecules (Bop1/Bop2) were used as negative and positive controls, respectively. Together, these results suggest that the capacity of β3-endonexin binding may correlate with the antiangiogenic function of mβCTPs. Thus, we further tested if the peptides of β3CTP and β5CTP can directly bind β3-endonexin. In the yeast 2-hybrid assay, we found that β3-endonexin did interact with β5CTP. However, the interacting signal between β3-endonexin and β3CTP was substantially weak (Fig. 6c), which is consistent with the finding that mβ5CTP is more effective than mβ3CTP in antitumor (Fig. 3). Nonetheless, results from a competition experiment showed that either mβ3CTP or mβ5CTP was able to inhibit the β3 CT binding to β3-endonexin (Fig. 6d), verifying that both β3CTP and β5CTP can interact with β3-endonexin.

### β3-Endonexin is required for HUVEC tube formation but not essential for integrin-mediated HUVEC adhesion and migration

**Fig. 7:**
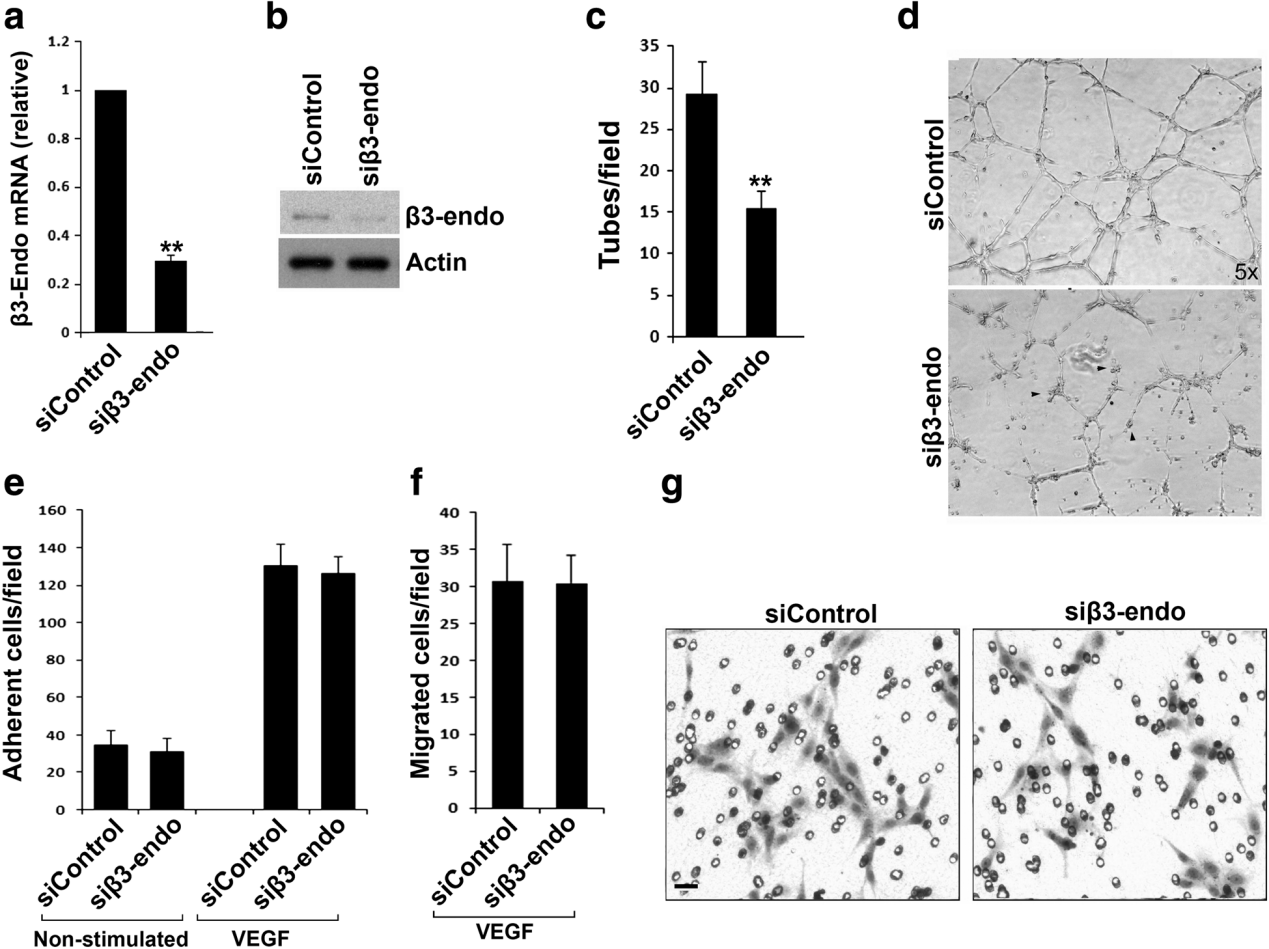
β3-Endonexin is able to promote HUVEC tube formation but not required for VEGF-induced HUVEC adhesion and migration. **a, b** HUVECs were transfected with an siRNA duplex specifically targeting β3-endonexin (siβ3-endo). A non-targeting siRNA duplex (siControl) was used as a control. Cells were harvested 48 h after transfection, and expression of β3-endonexin in HUVECs was quantified by qRT-PCR for mRNA (**a**) and western blotting for protein (**b**). **c, d** After transfected with siRNA for 48 h, HUVECs were harvested and used for the tube formation assays. The formed capillary-like structures were counted and the disconnected structures were indicated (arrow heads). **e-g** HUVECs transfected with either siControl or siβ3-endo were used in adhesion (**e**) and migration (**f, g**) assays in the absence and presence of VEGF. The adhered or migrated cells were quantified as described in methods. Results were expressed as means ±SD from three experiments; statistical significance was analyzed using Student’s *t* test (**, *p* < 0.01)

To test if β3-endonexin in vascular endothelia cells plays a role in angiogenesis, we used a specific siRNA to knock down endogenous β3-endonexin in HUVECs, and meanwhile used a non-targeting siRNA as a control. Transfection of HUVECs with the siRNA for β3-endonexin significantly suppressed the expression of β3-endonexin when compared with the control siRNA (Fig. 7a, b). Importantly, we found that HUVEC tube formation was significantly impaired when down-regulating endogenous β3-endonexin (Fig. 7c, d). This result suggests that endogenous β3-endonexin in vascular endothelial cells is required to support angiogenesis.

Since β3-endonexin has been identified as a binding partner for the integrin β3 CT, we further tested if β3-endonexin is involved in regulating αvβ3-mediated HUVEC adhesion and migration on fibrinogen. As shown in Fig. 7e-g, knockdown of endogenous β3-endonexin neither affected HUVEC adhesion in the presence or absence of VEGF stimulation, nor altered VEGF-induced HUVEC migration. These results suggest that β3-endonexin is dispensable for integrin-mediated HUVEC adhesion and migration, and also indicate that the proangiogenic role of β3-endonexin may be through an integrin-independent mechanism.

### Both the antiangiogenic mβCTPs and β3-endonexin are involved in regulating HUVEC proliferation and VEGF expression in HUVECs

**Fig. 8:**
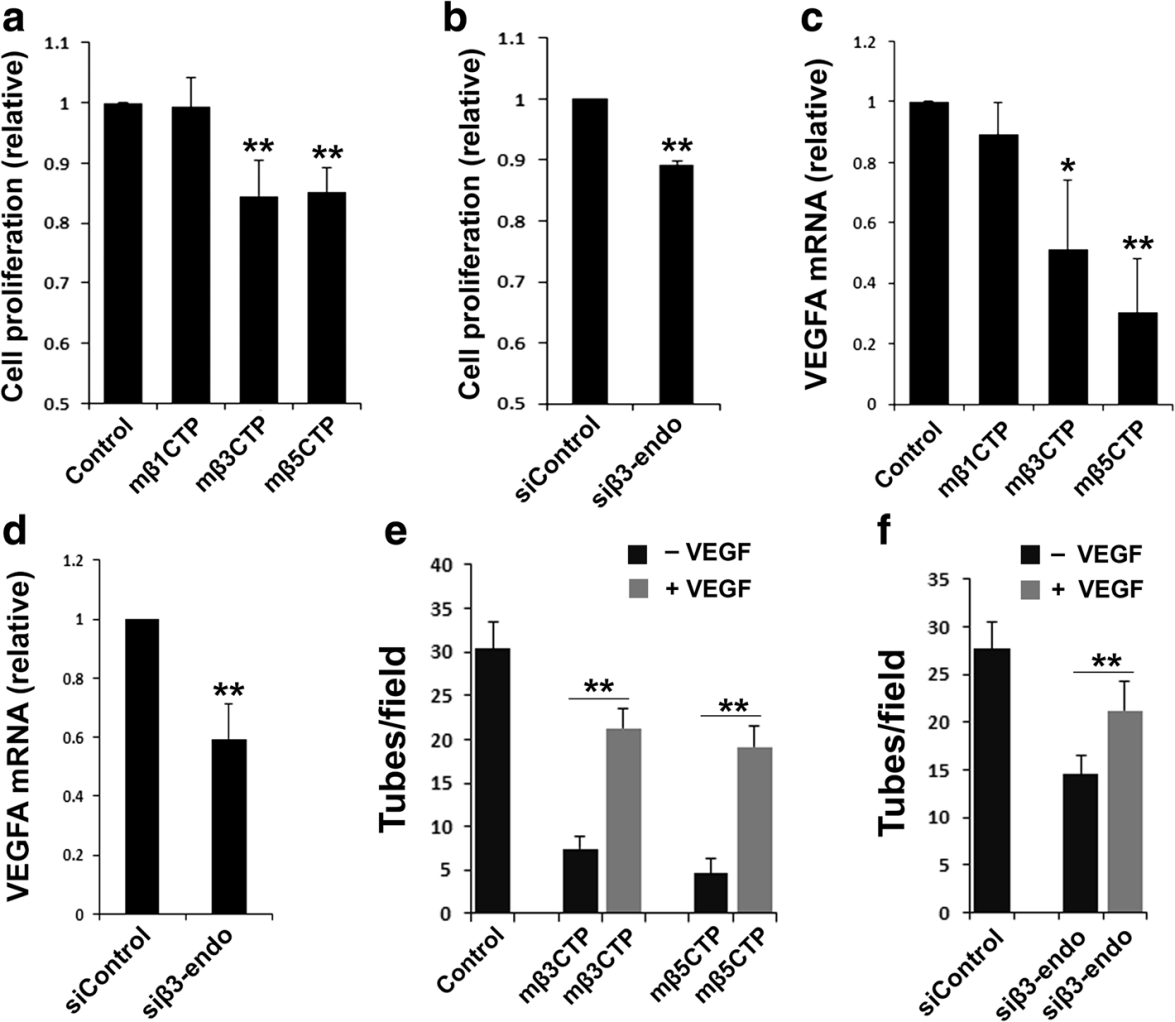
Treatment with the antiangiogenic mβCTPs and knockdown of endogenous β3-endonexin in HUVECs both suppress cell proliferation and VEGFA expression. HUVECs were treated with the indicated mβCTPs (20 μM at a final concentration) or transfected with siRNA (siControl or siβ3-endo). **a, b** HUVEC proliferation was evaluated using CCK-8 cell proliferation assay. **c, d** The expression levels of VEGFA mRNA in HUVECs were quantified by qRT-PCR. **e, f** HUVECs were treated with either siRNAs or mβCTPs as indicated and seeded on polymerized Matrigel in 24-well plates in the absence or presence of VEGF (20 ng/ml). The formed capillary-like structures were counted under an inverted microscope (5× objective). Results were expressed as means ±SD of three experiments; statistical significance was analyzed using Student’s *t* test (*, *p* < 0.05; **, *p* < 0.01)

To mechanistically understand the functional correlation between the antiangiogenic mβCTPs and β3-endonexin, we evaluated each of their influences on cell proliferation and VEGF expression in HUVECs, since it was reported that the β3-endonexin family is involved in regulating both events [24, 33]. We found that treatment of HUVECs with both mβ3CTP and mβ5CTP, two antiangiogenic peptides, significantly suppressed HUVEC proliferation, as evaluated by the CCK-8 Cell Counting Kit (Fig. 8a) and the MTT assay (Additional file 1: Figure S5). However, treating HUVECs with mβ1CTP, one of the non-antiangiogenic mβCTPs, failed to affect cell proliferation. In addition, treatment with both mβ3CTP and mβ5CTP but not with mβ1CTP was found to suppress the expression of VEGF in HUVECs (Fig. 8c). Consistently, knocking down endogenous β3-endonexin in HUVECs by siRNA was able to inhibit both cell proliferation and VEGF expression (Fig. 8b, d). Importantly, we found that addition of VEGF could alleviate the suppression of HUVEC tube formation by the antiangiogenic mβCTPs or siRNA against β3-endonexin (Fig. 8e, f), suggesting that the antiangiogenic effect of mβCTPs or siRNA against β3-endonexin may be due to their suppression on VEGF expression, at least partially. Taken all together, these results suggest that the antiangiogenic mβCTPs may directly bind to β3-endonexin and sequester its function in promoting VEGF expression in endothelial cells.

## Discussion

Integrins have become the attractive targets for developing antiangiogenic strategies to treat solid tumors for many years. Specifically, the expression of αvβ3 is significantly up-regulated in new born vessels, which makes αvβ3 an ideal antiangiogenic target [6, 34]. Importantly, preclinical studies have shown that some integrin αvβ3 antagonists targeting the extracellular ligand binding exhibit promising antiangiogenic effects [35]. Nonetheless, the antiangiogenic potential of the integrin CTs has not been well evaluated. Previously, we have observed that kindlin-2, one of the integrin β CT binding proteins and a key integrin activator, is required to support integrin αvβ3-mediated endothelial cell adhesion, migration and angiogenesis [20, 26]. In addition, we have identified a core region in the integrin β1 and β3 CTs for binding to kindlin-2, a 12 amino-acid sequence (βCTP) [25, 36]. Based on these finding, we designed a panel of membrane-permeable peptides (mβCTPs) by conjugating each one of the βCTPs with a cell penetrating peptide and tested their antiangiogenic function. Interestingly, we find that mβ3CTP and mβ5CTP can inhibit angiogenesis using both in vitro and in vivo models while mβ6CTP exhibits a moderate antiangiogenic capacity. However, the other mβCTPs, including mβ1CTP, mβ2CTP and mβ7CTP, do not show significant antiangiogenic ability. Therefore, the antiangiogenic ability of the mβCTPs is not consistent with the binding repertoire between the corresponding β CTs and kindlin-2. Further mapping the antiangiogenic core in mβ3CTP discloses that the C-terminal RGT residues in the β3 CT are not essential, although these residues (RGT) are critically required for β3 CT binding to kindlin-2 [37]. Hence, we conclude that the antiangiogenic function of mβCTPs is independent of their binding to kindlin-2.

In addition to kindlin-2, a few of other proteins have been found to be able to interact with the NITY motif in integrin β3 CT, such as Shc [38], Grb2 [32], myosin [39], Src [40–42] and β3-endonexin [21, 22]. While binding of the β3 CT to Shc, Grb2 and myosin requires tyrosine phosphorylation of the NITY motif, interaction of β3 CT with β3-endonexin and Src is independent of tyrosine phosphorylation [22, 42]. Interestingly, both β3-endonexin and Src can selectively bind the β3 CT, but not the β1 and β2 CTs, which is in line with the antiangiogenic feature of mβCTPs. However, the antiangiogenic core identified in mβ3CTP is only compatible with the β3-endonexin binding site in the β3 CT [41], highlighting that β3-endonexin, but not Src and others, may serve as an effector for the antiangiogenic mβCTPs.

β3-Endonexin, a 111 amino-acid polypeptide, has been found to be expressed in vascular endothelial cells [24, 27]. Interestingly, its expression has been found to be significantly up-regulated in post-chemotherapeutic lung cancer tissues [43], indicating that β3-endonexin might be involved in regulating angiogenesis. In our study, we demonstrate that β3-endonexin is proangiogenic and able to support HUVEC proliferation, tube formation and VEGF expression. However, a previous study reported that under hypoxic conditions β3-endonexin acted as an antiangiogenic factor [24]. The molecular mechanism underlying this discrepancy is not clear, but it might be owing to the different experimental conditions used in these two studies (normoxia vs. hypoxia). As a transcriptional cofactor, the regulation of β3-endonexin on target genes might be different under normoxia and hypoxia. Further studies, especially profiling the target genes of β3-endonexin under these conditions, should be helpful to delineate the mechanisms.

Since β3-endonexin is dispensable in integrin-mediated endothelial cell adhesion and migration, it may possibly employ an integrin-independent mechanism in supporting angiogenesis. We also performed the Co-IP assay using an anti-αvβ3 antibody and failed to co-precipitate endogenous β3-endonexin in endothelial cell lysates while endogenous kindlin-2 could be co-precipitated with integrins (not shown). The failure of detecting the interaction between β3-endonexin and integrins at the endogenous levels suggests that the integrin β CT in endothelial cells may be predominantly occupied by kindlin-2 (or other binding partners) but not β3-endonexin, possibly due to its lower binding affinity and/or expression levels. It might be more practical to enrich endogenous β3-endonexin in the Co-IP assay by using an anti-β3-endonexin antibody. Unfortunately, such an antibody that can be used to precipitate β3-endonexin in the cell lysate is currently unavailable in our laboratory. These observations somehow indicate that the proangiogenic effect of endogenous β3-endonexin is unlikely through its direct binding to integrins. Nonetheless, exogenous delivery of the β3-endonexin-binding mβCTPs to endothelial cells may effectively sequester endogenous β3-endonexin. And possibly, when the expression levels of endogenous integrins in vascular endothelial cells are significantly upregulated under certain pathological conditions, they may also be involved in binding to and sequestering endogenous β3-endonexin. If so, this may explain why enhanced tumor growth can be observed in mice with deficiency of the β3 integrins [12].

The β3-endonexin family also includes two other isoforms, β3-endonexin-L (170 amino-acids) and NRIF3 (177 amino-acids), both of which contain the 111 amino-acid β3-endonexin at their N-termini [21, 44]. β3-Endonexin-L and NRIF3 are almost identical except that the C-terminal 9 residues in β3-endonexin-L are replaced with a 15 amino-acid sequence in NRIF3. Interestingly, β3-endonexin-L does not bind the integrin β3 CT [21], suggesting that the β3 CT-binding site might be buried inside in the longer β3-endonexin isoforms by their C-terminal residues. Therefore, the antiangiogenic ability of the mβCTPs may not be relevant to the longer β3-endonexin isoforms.

It has been known that the NITY motif in the β3 CT is an essential binding site for β3-endonexin (Table 1) [21, 22]. In addition, the N-terminal residues proximal to the NITY motif also participate in the binding. Substantially, substitution of the NITY motif with NPTY blunts the binding of β3 CT to β3-endonexin, suggesting that the NPxY motif in the integrin β CTs may be not a favorable binding site for β3-endonexin. This may explain why only the integrin β3, β5 and β6 CTs, but not the β1, β2 and β7 CTs, interact with β3-endonexin. In fact, the binding ability of the β3, β5 and β6 CTs to β3-endonexin are not equal. Interestingly, it seems that the binding capacity determines the functional consequence. As we observed, mβ6CTP, derived from the β6 CT with a relatively weak binding to β3-endonexin, is also less effective in antiangiogenesis.

As a polypeptide carrying a nuclear localization sequence, β3-endonexin can localize in both the nucleus and cytoplasmic region of endothelial cells [27]. A previous study showed that β3-endonexin could regulate u-PAR promoter activity through the PEA3/ets binding site [45]. And interestingly, transcriptional factor PEA3 was demonstrated to be able to activate VEGF transcription [46]. Therefore, these findings indicate that β3-endonexin may participate in regulating VEGF expression by acting as a transcription cofactor of PEA3 (or some other unidentified ones). Besides, β3-endonexin may employ additional mechanisms to support angiogenesis since VEGF can only partially rescue the function (Fig. 8e, f). Hypothetically, the antiangiogenic mβCTPs may direct bind β3-endonexin and functionally block its interaction with the downstream targets (transcription factors or DNA sequences). Obviously, further studies are required to delineate the mechanisms.

## Conclusions

Taken together, our findings in this study disclose that certain of peptides derived from the integrin β CTs interact with β3-endonexin and also are antiangiogenic, revealing a novel antiangiogenic strategy. Since β3-endonexin in vascular endothelial cells promotes VEGF expression and cell proliferation, two essential proangiogenic events, we therefore conclude that the antiangiogenic mβCTPs may directly bind β3-endonexin and sequester its proangiogenic property in endothelial cells.

## Additional file

Additional file 1: **Figure S1.** mβ3CTP is membrane-permeable. The peptides of mβ3CTP (a) and β3CTP (b) were Nterminally conjugated with FITC and used to incubate with HUVECs, and the signal of FITC in HUVECs was evaluated under fluorescent microscopy. Scale bar, 15 μm. **Figure S2.** The antiangiogenic peptides do not suppress RM1 cancer cell proliferation. RM1 cancer cells were treated with mβ3CTP or mβ5CTP (each at 20 μM) and their effects on cell proliferation were evaluated using CCK-8 cell proliferation assay. Cells without treatment were used as a control. **Figure S3.** Both mβ3CTP and mβ5CTP suppress in vivo tumor growth. RM1 cancer cells were subcutaneously injected into BALB/c nude mice. Mice were treated with mβ3CTP and mβ5CTP as described in Methods. PBS alone was used as a control. Mice were sacrificed at the end point and tumor tissues were isolated and photographed. **Figure S4.** mβ3CTP and mβ5CTP fail to affect VEGF-induced HUVEC adhesion and migration on vitronectin. a HUVECs were treated with the indicated mβCTPs (20 μM) and allowed to adhere to immobilized vitronectin for 30 min in the absence or presence of VEGF (20 ng/ml). b HUVECs were treated with the indicated mβCTPs (20 μM) and allowed to migrate on Transwell membrane coated with vitronectin for 8 h in the presence of VEGF (20 ng/ml). The adhered and migrated cells were fixed, stained, photographed and counted (*, *p* < 0.05; **, *p* < 0.01). **Figure S5.** The antiangiogenic mβCTPs and siRNA against β3-endonexin suppress HUVEC proliferation. HUVECs were treated with the indicated mβCTPs (a) or siRNA against β3-endonexin (b). Their effects on cell proliferation were evaluated using the MTT assay (**, *p* < 0.01). (PDF 230 kb)

## Abbreviations

CT: Cytoplasmic tail
mβCTP: membrane-permeable β-integrin cytoplasmic tail peptide
VEGF: Vascular endothelial growth factor
βCTP: β-integrin cytoplasmic tail peptide
