# Supplementary material for: Peptides derived from the integrin β cytoplasmic tails inhibit angiogenesis
Source: Cell Commun Signal. 2018 Jul 3;16:38. doi: 10.1186/s12964-018-0248-8 (PMC6029062; doi:10.1186/s12964-018-0248-8)
Supplement: Supplementary file 1 — Figure S1. mβ3CTP is membrane-permeable. The peptides of mβ3CTP (a) and β3CTP (b) were Nterminally conjugated with FITC and used to incubate with HUVECs, and the signal of FITC in HUVECs was evaluated under fluorescent microscopy. Scale bar, 15 μm. Figure S2. The antiangiogenic peptides do not suppress RM1 cancer cell proliferation. RM1 cancer cells were treated with mβ3CTP or mβ5CTP (each at 20 μM) and their effects on cell proliferation were evaluated using CCK-8 cell proliferation assay. Cells without treatment were used as a control. Figure S3. Both mβ3CTP and mβ5CTP suppress in vivo tumor growth. RM1 cancer cells were subcutaneously injected into BALB/c nude mice. Mice were treated with mβ3CTP and mβ5CTP as described in Methods. PBS alone was used as a control. Mice were sacrificed at the end point and tumor tissues were isolated and photographed. Figure S4. mβ3CTP and mβ5CTP fail to affect VEGF-induced HUVEC adhesion and migration on vitronectin. a HUVECs were treated with the indicated mβCTPs (20 μM) and allowed to adhere to immobilized vitronectin for 30 min in the absence or presence of VEGF (20 ng/ml). b HUVECs were treated with the indicated mβCTPs (20 μM) and allowed to migrate on Transwell membrane coated with vitronectin for 8 h in the presence of VEGF (20 ng/ml). The adhered and migrated cells were fixed, stained, photographed and counted (*, p < 0.05; **, p < 0.01). Figure S5. The antiangiogenic mβCTPs and siRNA against β3-endonexin suppress HUVEC proliferation. HUVECs were treated with the indicated mβCTPs (a) or siRNA against β3-endonexin (b). Their effects on cell proliferation were evaluated using the MTT assay (**, p < 0.01). (PDF 230 kb) [file 12964_2018_248_MOESM1_ESM.pdf]

Supplemental Figures:

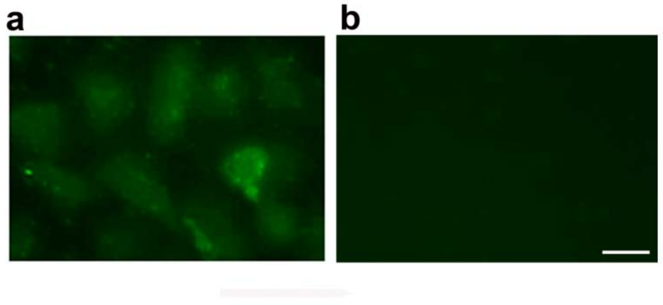

Figure S1. mβ3CTP is membrane-permeable. The peptides of mβ3CTP (**a**) and β3CTP (**b**) were N-terminally conjugated with FITC and used to incubate with HUVECs at a concentration of 20 µM for 2 hours. After washing, the signal of FITC in HUVECs was evaluated under fluorescent microscopy. Scale bar, 15 µm.

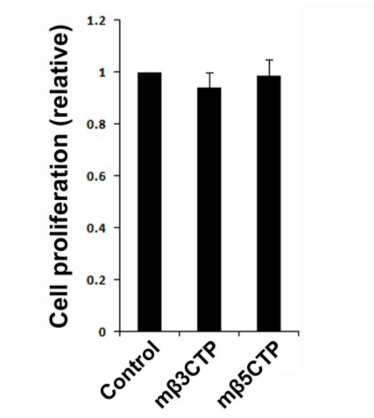

Figure S2. The antiangiogenic mβ3CTP and mβ5CTP do not suppress RM1 cancer cell proliferation. RM1 cancer cells were treated with mβ3CTP or mβ5CTP (each at 20 µM) and their effects on cell proliferation were evaluated using CCK-8 cell proliferation assay. Cells without treatment were used as a control.

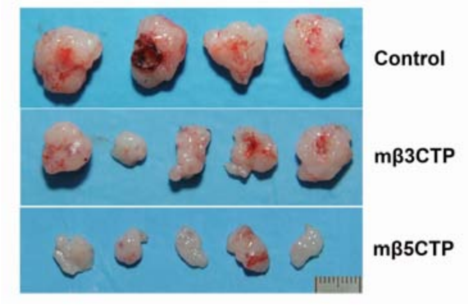

Figure S3. Both mβ3CTP and mβ5CTP suppress *in vivo* tumor growth. RM1 cancer cells were subcutaneously injected into BALB/c nude mice. Starting on day 5, the tumor areas were subjected to treatment by local injection of 100 µl of the indicated mβCTP solution (50 µM at a final concentration) every other day. PBS alone was used as a control. Mice were sacrificed at the end point and tumor tissues were isolated and photographed.

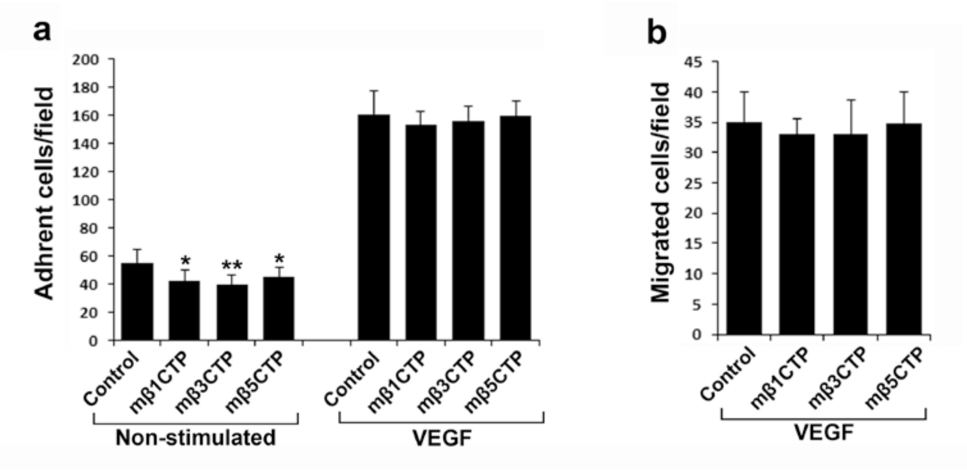

Figure S4: mβ3CTP and mβ5CTP fail to affect VEGF-induced HUVEC adhesion and migration on vitronectin. a HUVECs were treated with the indicated mβCTPs (20 µM) and allowed to adhere to immobilized vitronectin for 30 min in the absence or presence of VEGF(20 ng/ml). The adherent cells were fixed, stained and counted as described in methods. **b** HUVECs were treated with the indicated mβCTPs (20 µM) and allowed to migrate on Transwell membrane coated with vitronectin for 8 hours in the presence of VEGF (20 ng/ml). The migrated cells were fixed, stained, photographed and counted. Results were expressed as means ± SD of five samples; statistical significance was analyzed using Student's *t* test (*, $p < 0.05$; **, $p < 0.01$).

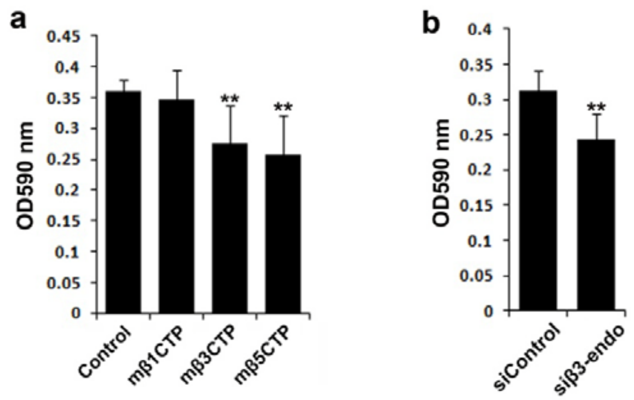

Figure S5. The antiangiogenic mβCTPs and siRNA against β3-endonexin suppress HUVEC proliferation. HUVECs were treated with the indicated mβCTPs (**a**) or siRNA against β3-endonexin (**b**). Their effects on cell proliferation were evaluated using the MTT assay. Results were expressed as means ± SD of five samples; statistical significance was analyzed using Student's *t* test (**, $p < 0.01$).
